# Modeling Radiofrequency Electromagnetic Field Wearable Distributed (Multi-Location) Measurements System for Evaluating Electromagnetic Hazards in the Work Environment

**DOI:** 10.3390/s25154607

**Published:** 2025-07-25

**Authors:** Krzysztof Gryz, Jolanta Karpowicz, Patryk Zradziński

**Affiliations:** Central Institute for Labour Protection—National Research Institute (CIOP-PIB), 00-701 Warszawa, Poland; krgry@ciop.pl (K.G.); jokar@ciop.pl (J.K.)

**Keywords:** radiofrequency electromagnetic fields, personal exposure meters, measurements, workers’ exposure

## Abstract

**Highlights:**

**What are the main findings?**

- The appropriate use of a distributed (multi-location) wearable measurement system may significantly improve the quality of exposure evaluation, i.e., reduce discrepancies between the values of the unperturbed radiofrequency (RF) electromagnetic field (EMF), which is considered in the standardized procedure of evaluating workers’ EMF exposure, and EMF measured by wearable equipment, which is impacted by the proximity of the body of worker wearing this equipment.
- It was confirmed that the helmet, which is mandatory in many work environments, is a potential location of EMF prob in such of distributed measurement system.

**What are the implications of the main findings?**

- Distributed wearable EMF measurement systems may allow the autonomous evaluation of workers’ exposure to RF EMF without continuous supervision of shift-long measurements by personnel responsible for evaluating this exposure, while maintaining a quality sufficient for the exposure evaluation required by labor law.
- Using the helmet routinely worn by workers, adapted to carry an EMF-sensitive probe and other electronics of the distributed measurement system, would not unduly increase the worker’s load, unlike another additional (not routine) outfit supporting EMF probes, which would need to be carried by anyone whose EMF exposure is evaluated.

**Abstract:**

The investigations examined a potential reduction in discrepancies between the values of the unperturbed radiofrequency (RF) electromagnetic field (EMF) and values of the EMF measured by wearable equipment (personal exposure meters) impacted by the proximity of the human body. This was done by modelling distributed wearable (multi-location, with up to seven simultaneously locations) measurements. The performed numerical simulations mimicked distributed measurements in 24 environmental exposure scenarios (recognized as virtual measurements) covered: the horizontal or vertical propagation of the EMF and electric field vector polarization corresponding to typical conditions of far-field exposure from wireless communication systems (at a frequency of 100–3600 MHz). Physical tests using three EMF probes for simultaneous measurements have been also performed. Studies showed that the discrepancy in assessing EMF exposure by an on-body equipment and the parameters of the unperturbed EMF in the location under inspection (mimicking the contribution to measurement uncertainty from the human body proximity) may be significantly reduced by the appropriate use of a distributed measurement system. The use of averaged values, from at least three simultaneous measurements at relevant locations on the body, may reduce the uncertainty approximately threefold.

## 1. Introduction

### 1.1. Sources of Environmental Radiofrequency EMF Exposure

Radiofrequency (RF) electromagnetic field (EMF) is commonly used by radio communication systems to transmit information wirelessly. The emitted RF EMF may provide a global range of data exchange (e.g., public mobile communication systems operating over long distances) or only local one (e.g., short range devices operating over distances of up to a several hundred meters, used in applications such as: Internet of Things (IoT), Radiofrequency Identification (RFID) and Wireless Local Area Network (WLAN) equipment) [1,2,3,4].

**Figure 1:**
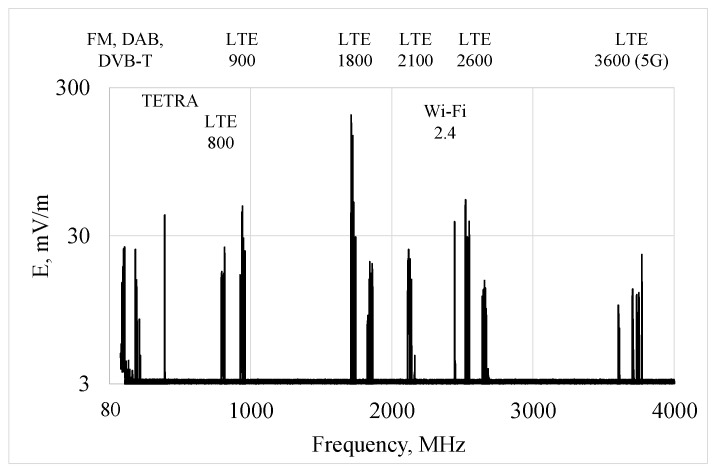
The RF EMF frequency spectrum recorded in the center of Warszawa, Poland, in 2025 [authors archive].

The area of application of wireless radio communication systems is constantly broadening, which is also increasing the number of RF EMF sources. Devices equipped with wireless functionality connect directly to each other (e.g., in the Wireless Fidelity (Wi-Fi) or Bluetooth systems) or through intermediating networks (e.g., in a network of mobile communication base stations or Wi-Fi access points) [1]. Where there are a large number of subscribers of communication services, e.g., in an urban environment, antennas of mobile communication systems with omni- or directional radiation patterns, which are designed for efficient signal transmission over long distances, are typically located on free-standing masts and roofs of buildings. It is estimated that 5G mobile communication technology enables support for as many as one million devices in an area of 1 km^2^ [5]. As a result of using radio communication systems and continuous electromagnetic emissions, in the environment of work and everyday life, virtually everyone is exposed to a RF EMF of complex frequency composition (Figure 1). The typical main radio communication sources of environmental exposure to RF EMF in Poland are characterized in Table 1. Taking into account the high degree of international standardizing in the field of radio communication, a similar frequency pattern of radio communication systems is in other countries.

Studies on the health impact of RF EMF emitted by wireless communication systems, which are discussed in the international literature, most often include exposure from mobile phone terminals (local exposure to the user’s body at a higher level of RF EMF and a shorter duration than exposure caused by the network of base stations). These studies showed that public exposure to RF EMF may affects brain activity and increase the risk of glioma and acoustic neuroma in the population of heavy users of mobile phones [7]. In most studies, the genotoxic effects of RF EMF exposure at non-thermal levels were not observed, although DNA breaks and mitotic spindle disorders were observed in some cases [7].

Because of harmful health effects (taking long-term and short-term exposure effects into account) various measures are applied to limit RF EMF exposure of workers and the general public. Directive 2013/35/EU requires that workers are protected against electromagnetic hazards [8]. However, in the European Union there are no mandatory requirements regarding protection of the general public against electromagnetic exposure—the Council of the European Union provided limits for general public exposure to EMF as non-mandatory recommendations only [9]. Most of the European Member States have adopted public exposure limits based on these recommendations or have included measures providing for more restrictive protection of the population [10].

Due to the ubiquitous presence of RF EMF sources, it is necessary to identify the sources and characteristics of emitted EMF and to assess the consequences of human exposure, especially in new areas of their use.

### 1.2. Measurements of EMF Exposure

**Figure 2:**
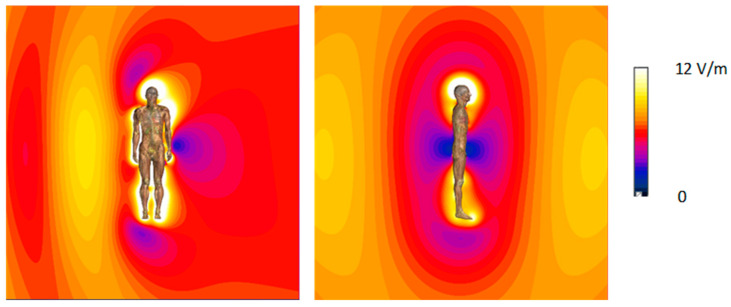
An example of cross-section (frontal and sagittal plane) of spatial distribution of the E-field near an insulated human body model for the exposure scenario: transverse direction of EMF propagation (from right to left of the model), vertical E-field polarization (from top to bottom), at 100 MHz frequency; linear scale; orange color applied to the level of 10 V/m [authors archive].

The criteria for assessing exposure to EMF provided by international safety guidelines refer to the unperturbed EMF (and evaluated omnidirectional) in the location under consideration [8,9,11,12,13,14]. Typically, standardized EMF measurements aimed at evaluating human RF EMF exposure are performed to determine the spatial distribution of the electric field (E-field) in the analyzed location, based on measurements using narrow- or broad- frequency band sensitive measurement equipment, typically employing small dimension ball-probes containing several E-field sensors to develop omnidirectional measurement results. Depending on the geometric configuration between the “human body—EMF meter—EMF source”, deformations of EMF spatial distribution may cause an increase or decrease in the EMF level, in the locations where the measurements are taken. This phenomenon is observed because human organisms have greater conductivity than the air and both the person whose exposure is being assessed and the person performing the measurements, may influence the spatial distribution of the evaluated EMF. Requirements for minimizing this impact of the human body, which can significantly increase the uncertainty of the EMF exposure evaluation, are specified in standardized protocols regarding environmental measurements, e.g., EN 50413 [15]. Usually, keeping some distance between the measurement device (EMF-sensitive probe) and the nearest human body is advised. Applying measures minimizing the impact of the human body on EMF distribution is particularly important in exposure cases, when the spatial distribution of RF EMF is highly dependent on the location of humans and technical objects in the area under question, such as in exposure to low-MHz range (Figure 2).

This standardized technique of evaluating EMF is particularly useful when EMF sources have a flat level of emission during the measurements, and when the location of the person whose exposure is being assessed is fixed towards EMF source. When workers are moving near EMF sources, assessing their exposure in the time domain is difficult. Determining the spatial distribution of EMF over a large area is also difficult. Personal exposure monitoring using on-body EMF recorders (data loggers) can provide more reliable measurement results in both cases.

### 1.3. On-Body Data Loggers in EMF Monitoring

However, for various reasons the variability of the EMF level affecting workers during their shift is also considered. The use of portable, battery-powered equipment with internal memories for recording large time series of measured EMF samples seems to be promising in this respect. Personal exposure meters (PEM), i.e., autonomous data loggers worn on the body, allow the exposure to EMF to be measured in terms of variability over time, i.e., EMF exposure time-pattern, which is caused by the activity of the worker who is wearing PEM and fluctuations in emissions from the source. The conditions of taking measurements using wearable PEMs significantly differ from the standardized methods of evaluating EMF exposure. The protocol of using wearable PEM should therefore specify how to compensate for specific factors causing the measurement results to differ from the parameters of the unperturbed EMF, which are subject to the legal limits set for the purposes of assessing human exposure [8,9,11,12,13,14].

Previous studies described in the research literature indicate that this technique is most often used in research regarding the characteristics of exposure to RF EMF emitted by radio communication systems [16,17]. The commercially available PEMs that are sensitive to RF EMF (more precisely E-field sensitive devices) allow broadband or narrowband (frequency selective) measurements of exposure caused by electromagnetic emissions from various wireless radio communication systems to be investigated [18]. The basic parameters of RF EMF data loggers commonly used for various research purposes are given in Table 2 and Table 3 [19,20,21,22]. All commercially available PEMs are designed for isotropic measurements with omnidirectional characteristic obtained by three axis-oriented internal RF EMF sensors. According to technical specifications, a deviation of isotropic PEMs sensitivity does not exceed 1.5 dB (19%).

Various researches performed using RF EMF data loggers, followed different measurement procedures, developed on an ad hoc basis for the needs of particular research projects, meaning that there is no unified approach in this area with common consensus [23,24,25,26]. RF EMF measurements with PEMs, especially narrowband (for example allowing the recognition of EMF sources), may bring various advantages over standardized measurements. However, the results of such measurements are affected by the significant impact from the proximity of the human body on the measured EMF, such as the “shielding effect” to the measured EMF caused by the body, or the anisotropy of the measurement probes (sensors) when the equipment is used in a wearable form [26,27].

The results of published research indicate that the use of single-location wearable RF EMF measurement equipment leads to an underestimation of exposure compared to what is measured by distributed multi-location equipment (for example, measurements with a twin-probe system, covering the operating frequencies of radio communication EMF sources, located at the front and back of the human body) [23,24,25]. In the case of commercially available equipment, this problem can be solved by using a cluster of several time-synchronized recorders, placed at various locations on the wearer’s body [28].

As mentioned, the procedures for using wearable PEMs have so far been arbitrary and different for individual research projects regarding monitoring exposure of the population. For example, in order to avoid the effect of shielding RF EMF by the human body, the E-field probes were placed on an insulating stick above the subject’s head [29]. The torso was assumed to be the primary relevant location for PEMs [23,24,25,26]. The limbs, neck and head were evaluated as having limitations for wearing regular EMF data loggers there, because their movement during measurements could affect the measurement results [23].

Another proposed method for limiting the influence of the human body on the measurement result of RF EMF is the calibration of measurement equipment in an anechoic chamber, when it is located on a human body [24]. In the studies on measuring environmental EMF disturbed by the body of a PEM wearer, the use of equipment calibrated in an anechoic chamber as an on-body wearable, allowed the error of exposure evaluation to be reduced to 3.2 dB (i.e., 1.5-times; 150%)—compared to 7 dB (i.e., 2.2-times; 220%)—estimating the case of using regular calibration of the measuring equipment in the unperturbed field (in an empty anechoic chamber).

### 1.4. The Aim of the Study

The aim of this study was to examine the efficiency of reducing the uncertainty of evaluating EMF exposure while employing wearable PEMs sensitive to RF EMF, through the use of a distributed (multi-location) measurement system, and subsequently evaluating exposure on the basis of the averaged results of measurements from sensors simultaneously worn on various body locations.

## 2. Materials and Methods

### 2.1. Virtual Measurements

When workers are at some distance from the EMF source, they receive EMF exposure at “far-field conditions”, which may justify the exposure evaluation being performed based on the E-field measurements [30]. In the case of “near-field conditions” which exist in proximity to the EMF sources, the evaluating of hazards from exposure is based on Specific Absorption Rate (SAR) being a measure of thermal effects, which denotes how much energy per unit time and unit mass is absorbed by the tissue.

**Figure 3:**
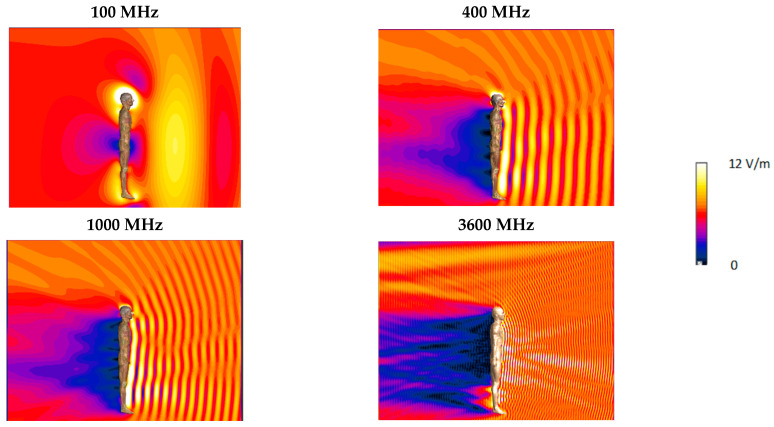
Sagittal cross-section of spatial distribution of the E-field near an insulated human body model: sagittal direction of EMF propagation (from front to back of the model), vertical E-field polarization (from top to bottom); linear scale; orange color applied at 10 V/m level.

The use of multi-location distributed RF EMF exposure evaluation was studied using virtual measurements mimicking a series of measurements in various far-field exposure scenarios through relevant numerical simulations. The investigations covered models of 24 exposure scenarios corresponding to typical exposure conditions in the environment, characterized by various directions of homogeneous RF EMF propagation with regard to the vertical axis of the human body model (Figure 3). The models of exposure scenarios represent various positions of the person against the EMF source through diverged RF EMF propagation: (i) transverse—from right to left side and E-field vertical; (ii) sagittal—from front to back and E-field vertical; (iii) vertical—from top to bottom and E-field horizontal perpendicular to the frontal cross-section; (iv) vertical—from top to bottom and E-field horizontal perpendicular to the sagittal cross-section. The simulations were repeated for typical frequencies of EMF emitted from different wireless radio communications systems: 100, 400, 700, 1000, 1800, 2600 and 3600 MHz, which cause usually far-field exposure in the urban environment as shown at Figure 1.

In the simulations, a constant value of 10 V/m of the E-field strength amplitude of continuous wave (CW) RF EMF was set at the edge of the analyzed area, from the boundary of space where the electromagnetic wave was radiated. It means, that the exposure scenarios showed the EMF distribution near the worker’s body exposed to RF EMF at the workplace, which would be evaluated at a level of 10 V/m of unperturbed EMF (i.e., in the absence of the worker at the workplace under inspection).

**Figure 4:**
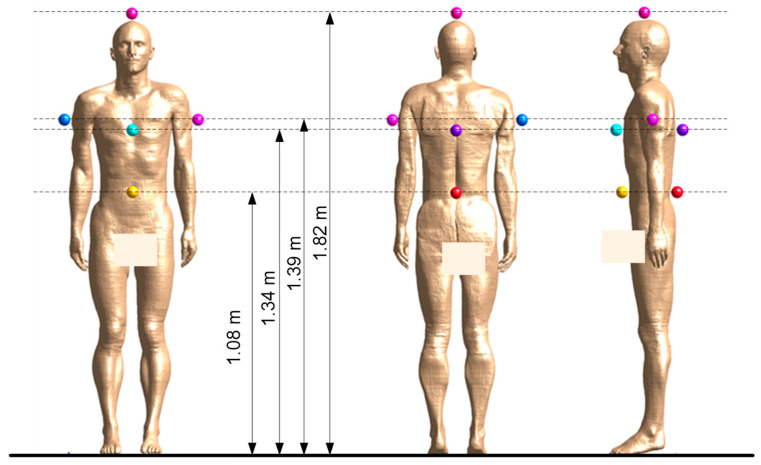
Location of E-field point assessment on waist (W), arms (A), chest (C) and head (H); view on the front, back and left side of the Duke body model.

On the basis of the results of relevant calculations regarding RF EMF spatial distribution, the E-field strength was determined at seven locations near the exposed body model: 5–55 mm away from the front (F), back (B), left and right sides (L and R) and top of the body model (H), for horizontal cross-sections at heights: 1.08 m for the waist (W) from the front and back; 1.34 m for the chest (C) from the front and back; 1.39 m for the right and left arms (A); and 1.82 cm for the head (H) (Figure 4). The locations of the analysed measurement points were selected to take into account the locations of PEMs which may be considered for the work environment measurements. As mentioned, for the measurements taken with research projects on public exposure in mind, PEMs were located in different places on body without limitations on where. However, in work environments potential PEM locations have to respect significant limitations—they cannot hinder the normal activities of the exposed worker and cannot cause any safety hazards in the work environment. For example, a location at the side of the waist may hinder the free hanging of the arms, while a location on the shoulder may increase the body’s outline and cause related discomfort or hazards for workers.

It was assumed that the applied locations of point-assessment of the E-field in the numerical simulations would mimic the results of real measurements from wearable equipment with E-field probe with a geometrical center in a considered location near the human body.

The calculated E-field strength was averaged over the volume of a sphere with a 50 mm diameter, as the dimensions of the simulated measurement probes. Manufacturers usually do not provide data on the exact dimensions of the measuring sensors in PEMs. The 50 mm diameter for simulated E-field averaging at the sensors location and probes of other equipment used in the environmental EMF measurements was chosen arbitrarily, taking into account the actual dimensions of various equipment. Additionally the point values of E-field strength simulated at distance 5, 10, 15, 20 mm to the body were determined for analyzed locations of the ball–probes measurement—to model the potential use of future small–dimension sensors in distributed measurement system.

The numerical simulations were performed using a finite-difference time-domain (FDTD) solver of Sim4Life software v7.2.4 (Zurich Med Tech, Zurich, Switzerland) and an anatomical male model (Duke v3.1—age: 34 years old, height: 1.77 m, weight: 70.2 kg, BMI ratio: 22.4, resolution: 2 × 2 × 2 mm, mapping of over 300 structures, organs and tissues of the human body). Duke’s anthropometric properties are close to the 50th percentile of male population used in ICRP 110 Adult Reference Computational Phantoms [31]. Dielectric parameters and densities of particular body tissues, of which the Duke numerical model is composed of investigated frequencies, were provided from the IT’IS Database for thermal and electromagnetic parameters of biological tissues based, among others, on the widely recognized and used data developed by Gabriel et al. [32,33]. The model was placed in a free space (analyzed area with a volume of 4 × 4 × 4 m) with boundaries at a 1.7–1.9 m horizontal distance from the body model, and a 1.1 m vertical distance from the top and the bottom in the case of the model insulated from the ground.

The numerical models of exposure scenarios were composed of approximately 510 million voxels, with the dimensions of a single voxel in the vicinity of the human body model in which the distribution of EMF was analyzed being 5 × 5 × 5 mm, which met the requirement of the EN IEC 62232 standard for this type of numerical calculations, which says that the voxel size should not be larger than 1/10 of the wavelength (for a maximum considered frequency of 3.6 GHz, the wavelength in air is approximately 83 mm) [30]. Convergence level (CL), which is quantity of accuracy of simulation and defined as the difference between the last two estimations in the frequency domain, was selected −50 dB (the medium in the used software settings).

### 2.2. Physical Measurements Using Multi-Location On-Body RF EMF Measurements System

**Figure 5:**
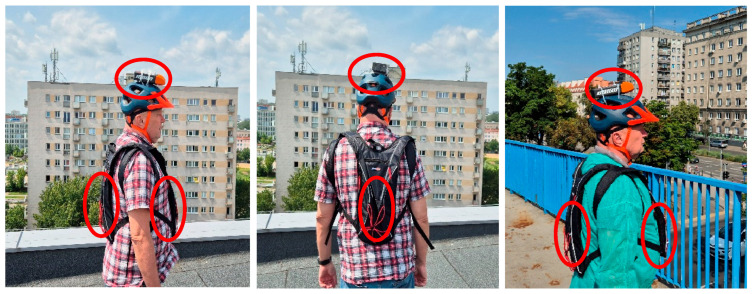
The locations of three PEMs, constituting the physical model of the multi-location on-body RF EMF measurement system, on the body of the researcher taking test measurements and the view to base station antennas emitting measured EMF (red circles: the location of PEMs).

The protocol of multi-location distributed RF EMF exposure evaluation was also self-tested using a physical model. Relevant measurements were performed in two real scenarios of exposure to RF EMF emitted by antennas of the public mobile communication system: (1) on the roof of a building with a height similar to the height of the antennas, where quasi-horizontal propagation of RF EMF may be assumed), (2) on a pedestrian bridge over a street, below the antennas, where quasi-vertical propagation of RF EMF may be assumed (Figure 5). The RF EMF frequency composition in the location of these tests was studied using a frequency spectrum analyser SRM-3006 (measurement range 0.2 mV/m–200 V/m in the frequency band 420–6000 MHz, manufacturer Narda, Pfullingen, Germany). The following components of recorded RF EMF, emitted from nearby base station antennas, were found: LTE800 (DL), LTE900 (DL), LTE1800 (DL), LTE2100 (DL), LTE2600 (DL & TDD) and LTE3600 (5G) (TDD)—frequency bands characterized in Table 1.

Measurements covered the time-synchronised recordings of the root-mean-square (RMS) value of the E-field strength in frequency bands compliant with the above-mentioned RF EMF frequency components found in the location of the tests. The physical model of a multi-location on-body measurement system was developed using three frequency-selective, narrowband PEMs of a type EME SPY Evolution (characterized in Table 3). Measurements were performed using a sampling rate of three seconds.

The sensitivity of each data logger was checked in the TEST-SAFE-BIO/CIOP-PIB calibration laboratory (accredited by the Polish Centre for Accreditation at EN ISO/IEC 17025 standard [34]/certificate No AP061)—in the continuous sinusoidal reference EMF produced inside the Gigahertz Transverse Electromagnetic Mode (GTEM) chamber, at frequencies relevant to the components of RF EMF in the location of the test measurements. The verification of the PEMS directional sensitivity was carried out using 3 orthogonal PEMs positions inside GTEM chamber. Individual correction factors C_fb_ for each PEMs in the particular frequency band (fb) were determined as average values of correction factors (ratio of E-field applied to E-field measured), obtained during measurements for 3 orthogonal position of PEM). C_fb_ factors were tested in each fb considered in this study: at center frequency from the bands used by public mobile communication and Internet access systems given in Table 1.

The PEMs were carried by a researcher (co-author of this study taking measurements) fixed in the backpacks (front and back locations of PEMs on the body), and the third PEM was fixed on the top of a plastic helmet. During the 10-min tests, the researcher performed slow rotations to the right and left in a defined measuring location, with the body in a straight position.

In the position where the vertical axis of the researcher’s body was during the test measurements, a vertical distribution of the unperturbed E-field was also determined by spot measurements without a presence of a researcher there. This was done for each of the frequency components of RF EMF present there, using the SRM-3006 analyzer.

## 3. Results and Discussion

### 3.1. Single Location On-Body RF-EMF Exposure Evaluation (Virtual Measurements)

For each of the 24 considered exposure scenarios (differing in RF EMF propagation direction, E-field vector polarization and frequency), the deviation in the results of the exposure assessment by a wearable device compared to the unperturbed EMF value was assessed based on the ratio of the E-field calculated in the particular location of the modeled E-field sensor (probe) to the unperturbed E-field value, set at the level of 10 V/m in simulated exposure scenarios. The following KX ratio is a measure of the influence from the proximity of the human body on the distribution of the evaluated E-field across all 24 exposure scenarios:

KX = E_x_/E_up_, (1)

where: E_x_—E-field value affecting location of an on-body single probe equipment; E_up_—the unperturbed E-field value set in the modeled exposure scenario (E_up_ = 10 V/m).

**Figure 6:**
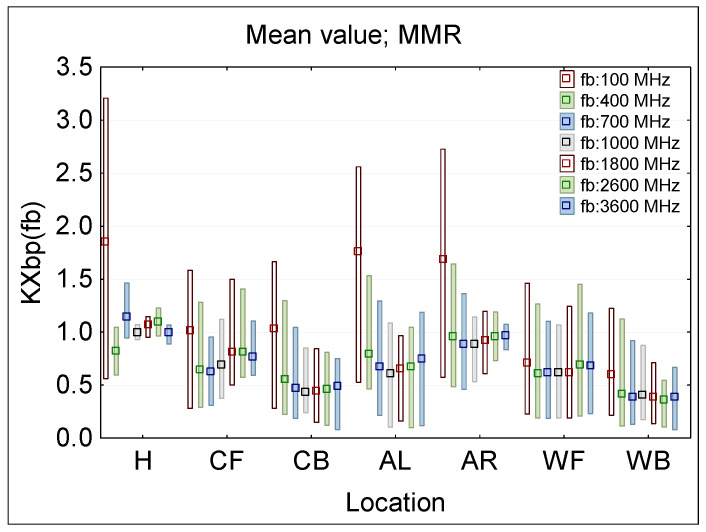
The KXbp(fb) ratio, presenting the discrepancy between the unperturbed E-field and the results of E-field measurements by multi-channel (narrowband) wearable equipment in various locations near the human body (analyzed subsets of results over all directions of RF EMF propagation and E-field vector polarizations, when the measurement equipment is located on: H—head, C—chest, A—arm, W—waist, F—front, B—back, R—right side, L—left side); the distribution of results at a particular frequency are characterized by MMR—the range from minimum to maximum values (bar), and mean value (dot).

**Figure 7:**
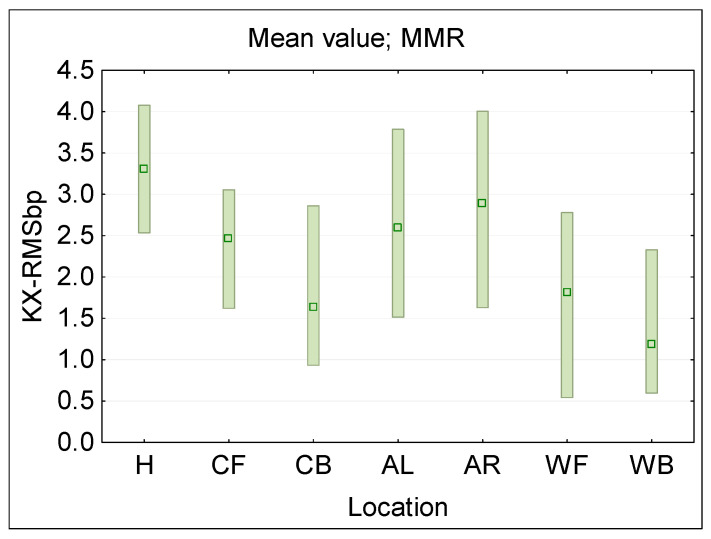
The KX-RMSbp ratio, presenting the discrepancy between the unperturbed E-field and the results of E-field measurements by broadband wearable equipment in various locations near the human body (analyzed subsets of results over all directions of RF EMF propagation and E-field vector polarizations, when the measurement equipment is located on: H—head, C—chest, A—arm, W—waist, F—front, B—back, R—right side, L—left side); the distribution of the broadband RMS values at a particular locations are characterized by MMR—the range from minimum to maximum values (bar), and mean value (dot).

Figure 6 presents the statistical analysis of KXbp(fb) evaluated following Formula (1) for each of a single ϕ50 mm E-field ball–probe (bp) location (sensor locations: H—head, C—chest, A—arm, W—waist, F—front, B—back, R—right side, L—left side), across the considered RF EMF frequencies (fb), as when evaluated using multi-channel narrowband PEM (MMR—minimum-maximum ranges and mean values). Figure 7 presents a distribution of broadband RMS value derived for each measurement location from the frequency selective results, modeling a broadband measurements following the formula:

(2) $$\mathrm{KX-RMSbp}\;=\;\sqrt{\sum {KXbp(fb)}^{2}}$$

where: KXbp(fb)—KX ratio established according to Formula (1) in analysed frequency band.

The largest dispersion of results (KXbp ratio), regardless of the single-point ball-probe measurement location, was obtained at the 100 MHz frequency—range from KXbp = 0.22 (for WB location) to KXbp = 3.21 (for H location). When the 100 MHz frequency, representing EMF exposure from FM radio broadcasting antennas, is excluded from the considerations, because of the limited number of location near such EMF sources, the smallest dispersion of results was found for the ball–probe measurement point located on the top of the head (H)—range from KXbp = 0.59 (for 400 MHz) to KXbp = 1.47 (for 700 MHz). However, performing measurements in real conditions with today’s regular equipment placed on the top of the head, e.g., on a helmet, may not be an acceptable solution for ergonomic reasons. Wearing them for many hours during measurement sessions over an entire shift may cause significantly increased fatigue to workers whose exposure is being assessed, e.g., when the equipment weighs more than double the weight of a typical helmet of mandatory use in the work environment, i.e., 300–450 g. The weight of commercially available devices ranges from 90 to 500 g, which may make it possible to arrange measurements using PEM on the helmet (using smaller PEM is better option), even without next generation of small equipment specially designed for such measurement protocol.

When a single-point ball-probe measurements do not include the head position (without H exposure scenarios), the maximum range of KXbp ratio variations is larger—from KXbp = 0.08 (for CB and WB at 3600 MHz) to KXbp = 2.73 (for AR at 100 MHz). When exposure scenarios regarding 100 MHz frequency are excluded, the variations obtained from the studied cases are from KXbp = 0.08 (for CB and WB at 3600 MHz) to KXbp = 1.64 (for AR at 400 MHz). In principle exposure evaluation by PEMs in such scenarios tends to be underestimated.

### 3.2. Multi-Location On-Body RF EMF Exposure Evaluation (Virtual Measurements)

**Figure 8:**
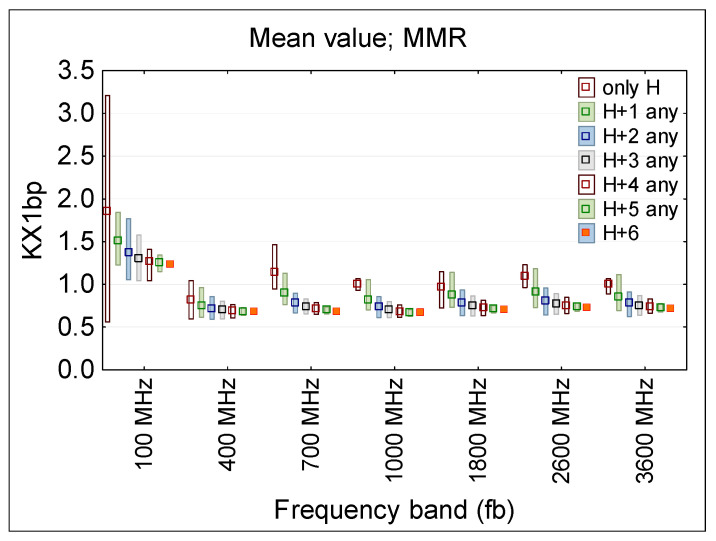
The results of multi-location ball-probe measurements (multi-channel narrowband analysis) evaluating the E-field exposure, normalized to the parameters of an unperturbed (homogeneous) E-field—the KX1bp ratio—in the location of the human body under exposure evaluation, averaged over all the considered exposure scenarios (two to seven ball-probes): measurement points located over the head (H), on the front or back of the chest or waist (CB, CF, WF, WB), and on the left or right arm (AL, AR); the distribution of results of KX1bp ratio is characterized by MMR—the range from minimum to maximum values (bar), and mean value (dot).

In articles presenting research using several PEMs [15,16,17,20] it was already suggested that limiting the dispersion of RF EMF on-body measurement results can be achieved by conducting wearable measurements simultaneously at several on-body locations. As mentioned earlier, in realistic measurement conditions, RF EMF sensitive equipment are usually placed on the torso, avoiding the head and arms, the movement of which may affect the operation of the devices [15]. Figure 8 (regarding narrowband multichannel measurements case) presents the KX1bp ratio (means and MMR values) derived following the formula:

KX1 = E_x1_/E_up_, (3)

where: E_x1_—E-field value measured by an on-body multi-location equipment in considered combination of locations: H, or H and combination of C, W and A; E_up_—the unperturbed E-field value set in the simulations (E_up_ = 10 V/m).

When using multi-location measuring equipment, the KX1bp ratio distribution may be evaluated from results of measurements obtained at various locations of the measurement ball-probes and averaged over any of two to seven considered locations of multi-point measurements: over the head (H), on the chest (C), waist (W) and arms (A).

**Figure 9:**
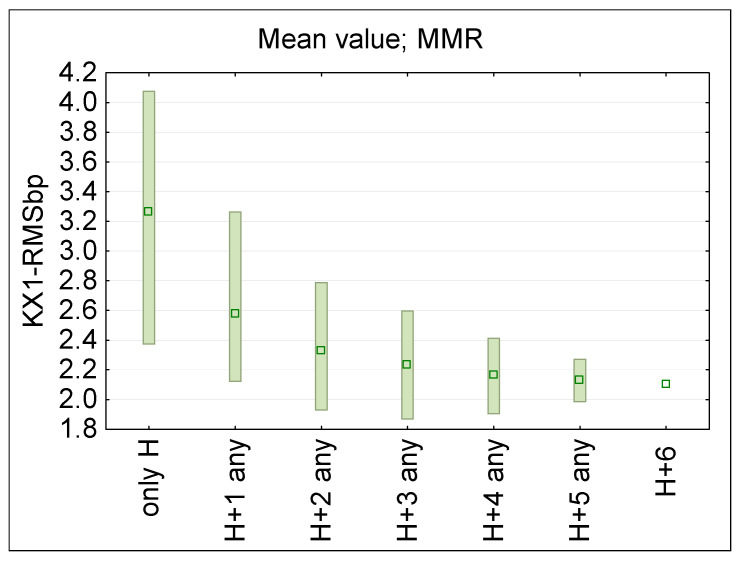
The results of multi-location measurements (broadband analysis) evaluating the E-field exposure, normalized to the parameters of an unperturbed (homogeneous) E-field—the KX1-RMSbp ratio—in the location of the human body under exposure evaluation, averaged for all the considered exposure scenarios: two to seven ball-probe measurement points located over the head (H), on the back or front of the chest or waist (CB, CF, WF, WB and on the left or right arm (AL, AR); the distribution of the broadband RMS values KX1-RMSbp are characterized by MMR—the range from minimum to maximum values (bar), and mean value (dot).

Figure 9 summarized KX1bp ratio evaluated for considered PEM locations when broadband measurements are performed using ball-probe (KX1-RMSbp).

Table 4, Table 5 and Table 6 compared the dispersion of results for single and multi-point measurement locations expressed by above mentioned KX1bp ratio across the considered RF EMF frequencies for ϕ50 mm E-field ball-probe, and KX1ss ratio in case of point E-field small-sensors located at various distances to body.

The largest differences, regardless of the number of measurement points covered by simultaneous measurements, were obtained for a frequency of 100 MHz (when the wavelength of EMF, approximately three meters there, is nearest to the human body height). At higher frequencies (with corresponding wavelength approximately 10–35 cm) the ranges of changes in the KX1bp ratios are smaller. This can be explained by the fact that at the 100 MHz frequency the so called “antenna effect”, which occurs the most significantly when the body height matches half of the wavelength—e.g., for height of Duke model 1.77 m, it is a 84.7 MHz—is still significant.

The obtained data confirms the hypothesis that increasing the number of simultaneous on-body measurement points, and using the averaged results from such measurements, significantly reduces the uncertainty of assessing workers’ RF EMF exposure. For example, increasing the measurement points from 2 to 3 reduces dispersion of measurements results (KX1bp and KX1ss ratios) by approx. 30–40% (Figure 8 and Figure 9). A smaller dispersion of measurement results was also obtained when omitting the location of measurement points on the arms (Figure 6 and Figure 7).

Additionally, the underestimation of the exposure level using the average value of the multi-point measurement on the human body in relation to the unperturbed RF EMF (which must be considered in legal evaluations of workers’ exposure), was found at a nearly flat level over the frequency range 400–3600 MHz (Figure 6 and Figure 8). Such a systematic underestimation of the exposure level may be compensated for by applying the relevant correction factors. In the considered exposure scenarios, a compensation factor of two may be derived from discussed study (Figure 8, Table 4). However, further studies need to be performed covering a broader set of exposure scenarios and experimental tests in the real work environment.

The changes in the distance of measurement points, simulated as small-sensor from the body do not cause significant changes in the distribution of KX1ss ratios (approx. 10–30%). The distance of the measurement probes from the body is therefore not a critical factor influencing the accuracy of exposure assessment using on-body measurement equipment.

### 3.3. Multi-Location On-Body RF EMF Exposure Evaluation (Physical Measurements)

The results of recordings in LTE800, LTE900, LTE1800, LTE2100, LTE2600 and LTE3600 (5G) frequency bands, collected by a three wearable PEMs following the two test measurements exposure scenarios described in Section 2.2, were compared with the unperturbed E-field value, which was measured in the location of the test measurements in each of the mentioned frequency bands without presence of anyone there.

The discrepancy in the sensitivity of the equipment used was taken into account by applying a relevant correction factor—C_f_ (covering discrepancy in the sensitivity of particular PEM and deviation of sensitivity from the omnidirectional one)—determined by the calibration of the PEMs in the laboratory reference RF EMF. The recordings from each PEM were normalized to the sensitivity unified across all the PEMs. Finally, the recordings were normalized to the level of the unperturbed E-field in the environment under study.

The following KSL ratio is a measure of the influence from the proximity of the human body on the distribution of the evaluated E-field during test measurements:

KSL = (C_fb_ × E_x_(fb))/E_up_(fb), (4)

where: C_fb_—individual correction factors determined for each used PEM to compensate for discrepancies in sensitivity between devices, performed separately in each analysed frequency band (fb); E_x_(fb)—the E-field value recorded in the analysed frequency band by an on-body PEM; E_up_(fb)—the unperturbed E-field value measured at particular fb during test measurements using omnidirectional spot ball-probe.

**Figure 10:**
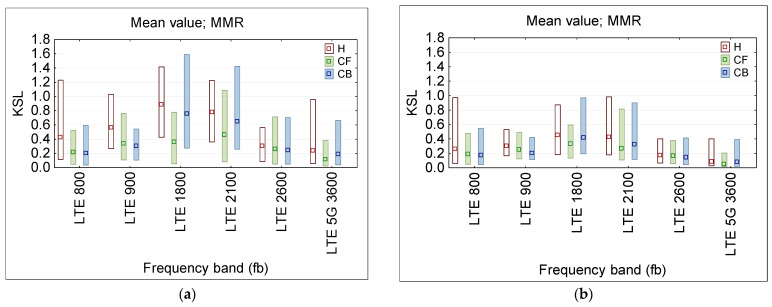
The KSL ratio, presenting the discrepancy between the unperturbed E-field and the results of recordings of the E-field influenced by the proximity of the body during the physical test measurements, to model multi-channel (narrowband) wearable equipment at various locations of single probes on the body, where particular PEMs were located: H—head, CF—chest font, CB—chest back): (**a**) 1st exposure scenario with quasi-horizontal EMF propagation; (**b**) 2nd exposure scenario with quasi-vertical EMF propagation; the distribution of results at a particular frequency band are characterized by MMR—the range from minimum to maximum of recorded values (bar), and the mean value (dot).

Figure 10 presents the statistical analysis (mean and MMR values) of this KSL ratio for each PEM, considered to be three on-body single-point measurements (E-field sensor locations: H—head, CF—chest front, CB—chest back). The smaller dispersion of results recordings was obtained in second exposure scenario with quasi-vertical RF EMF propagation (more typical in the urban out-door environment).

The reduction in the uncertainty of the measurement results caused by the impact from the proximity of the body (on the distribution of the evaluated E-field during the test measurements), when to the on-body distributed (multi-location) PEM recordings is applied, was analysed using the following KDM ratio:

KDM = E_dx_(fb)/E_up_(fb), (5)

where: (E_dx_(fb)—the arithmetic mean of the E-field value recorded in the analysed frequency bands by the on-body PEMs used, which constitute the modelled distributed measurement system, where each individual recording is normalized to compensate for any discrepancy in the sensitivity between devices following the formula: (C_fb_ × E_x_(fb)) (see Equation (4)), for each PEM used, performed separately for each analysed frequency band (fb); E_up_(fb)—the unperturbed E-field value measured at particular fb during the test measurements.

**Figure 11:**
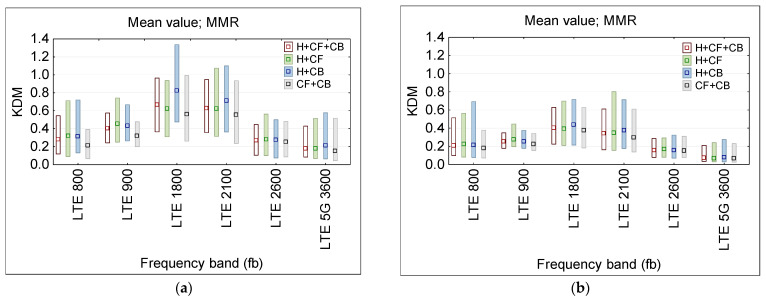
The KDM ratio, presenting the discrepancy between the unperturbed E-field and the results of recordings of the E-field influenced by the proximity of the body, during the physical test measurements, to model distributed on-body measurement system by a combination of various locations of single multi-channel (narrowband) wearable probes on the body, where particular PEMs were located at: H—head, CF—chest front, CB—chest back): (**a**) 1st exposure scenario with quasi-horizontal EMF propagation; (**b**) 2nd exposure scenario with quasi-vertical propagation; the distribution of results in a particular frequency band are characterized by MMR—the range from minimum to maximum of the recorded values (bar), and the mean value (dot).

Figure 11 presents the statistical analysis (MMR—mean and range between minimum and maximum values) of this KDM ratio for considering the particular combination of PEM locations (H—head and CF—chest front and CB—chest back), obtained during physical tests (note RF EMF from 100 and 400 MHz frequency bands was not present in the location of tests—what is very common in an urban environment).

The obtained data (Figure 11) confirm the conclusions from virtual measurements, whereby increasing the number of on-body measurement points (up to three points H + CF + CB case) reduces the difference between exposure evaluation results in exposure scenarios (a) and (b) of different EMF propagation and reduces the uncertainty of assessing workers’ RF EMF exposure (comparing to twin-points measurements) to the extend depending on the frequency and on-body location of particular measurement points up to 40%.

### 3.4. Distributed Measurement System Design

The major achievements of our work cover: considerations on a practical and important problem in occupational EMF exposure assessment using autonomous measurement systems; combining both numerical simulations and experimental validation of the studied cases to solve this problem using distributed multi-probes measurements; considerations covering comprehensive frequency range (100–3600 MHz) relevant to modern wireless systems; including statistical analysis of uncertainty reduction with multiple sensors developing practical recommendations for implementation of our conclusions in work environments.

Major limitations of our study, which next steps are planned, consist in: limited coverage of numerical modelling using a single body model only; the considered exposure scenarios covering the far-field exposure scenarios only with preliminary experimental validation in two real exposure scenarios and with slow movements only; only brief suggestions regarding directions of practical implementation solving potential challenges in the real work environment

Based on discussed research results and broader experience with various measurement techniques applied in evaluating EMF exposure in the work environment, considered practical problem may be summarize with suggestions on the structure and application of the multi-location measurement system designed for autonomous evaluation of workers exposure to RF EMF [35,36]:

- distributed measurement system composed of 3 autonomous (battery supplied) omnidirectional probes sensitive to E-field at relevant frequency bands
- autonomous data logger ensuring the synchronised sampling of RMS E-field strength from all probes, with at least 1 Hz sampling frequency and sampling synchronization better than 10% of sampling rate
- wireless communication between data logger and probes (to avoid safety hazards caused by cables connections of the measurements system components) or autonomous data logger in each probe
- the weight and dimensions of data logger and probes low enough to allow data-logger and probe#1 to be fixed into the regular helmet required to be used by worker in many work environments (or “measurement-helmet” structure designed to be the core of the distributed measurements system and used by worker instead of the regular one)
- the minimum capacity for the supplying system ensuring at least a shift-long autonomous measurement duration
- the shape, size and weights of probes#2 and #3 suitable to carry (wear) them in the locations discussed in this paper, e.g., in pockets of workers’ clothing (such as pockets of the special “measurement-vest”)
- the calibration and electromagnetic immunity of measurement system as required for the work place measurements (e.g., [37,38])

## 4. Conclusions

The results of our study show that the uncertainty of EMF exposure assessments (normalized to the parameters of the unperturbed EMF) from wearable RF EMF measurement equipment (delivering the results of measurements of parameters of EMF disturbed by the proximity of the user wearing this equipment) may be significantly reduced by appropriate using a distributed (multi-location) measurement system. This would allow workers’ exposure evaluating to be significantly improved using wearable measurements with respect to exposure limits set by labour regulations with regard to the parameters of unperturbed RF EMF.

The obtained results (mimicking the simultaneous application of two to seven measurements) suggest that this approach to evaluating workers’ RF EMF exposure may be sufficiently standardized for use in legally oriented studies.

The distributed wearable EMF measurements systems may allow the autonomous evaluation of workers’ exposure to RF EMF without continuous supervision of shift-long measurements by personnel responsible for evaluating workers’ exposure, while maintaining the quality sufficient for their results to be used in the exposure evaluation required by labor law.

It was confirmed the applicability of the helmet, which must be used in many work environments, as a potential location of one of measurement sensors in the distributed measurement system designed for the use in the work environment to improve the exposure evaluation quality. Using the helmet routinely worn by workers, adapted to carry an EMF-sensitive probe and other electronics of the distributed measurement system, would not un-duly increase the worker’s load, unlike another additional (not routine) outfit, which otherwise would need to be carried by the worker whose EMF exposure is evaluated.

Further studies are necessary to achieve broader parameterization of the analysed problems involving the uncertainty of EMF exposure evaluation using on-body measurement systems and the relevant procedures of analysing the measurement results.

## Figures and Tables

**Table 1:** Radio communication systems used in Poland [6].

RF EMF Sources	Frequency Band, MHz
Radio transmitters: analogue FM (*Frequency Modulation*) and digital DAB + (*Digital Audio Broadcasting*)	88–108 and 176–225
DVB-T (*Digital Video Broadcasting-Terrestrial*) TV transmitters	174–694
TETRA (*Terrestrial Trunked Radio*) digital mobile communication system	380–400
LTE (*Long Term Evolution*)—public mobile and Internet access systems:	
LTE700 (5G) ^1^	694–790
LTE800: DL and UL ^2^	791–821 and 832–862
LTE900: UL and DL	876–915 and 921–960
LTE1800: UL and DL	1710–1785 and 1805–1880
LTE2100: UL and DL	1920–1980 and 2110–2170
LTE2600: UL and DL	2500–2570 and 2620–2690
LTE2600 ^1^	2570–2620
LTE3600 (5G) ^1^	3400–3800
WiMAX (*Worldwide Interoperability for Microwave Access*) broadband Internet access systems ^1^	3600–3800
Wi–Fi (*Wireless Fidelity*)—local area network connectivity between devices and Internet access systems: Wi–Fi 2.4 GHz (used also by Bluetooth devices) ^1^ Wi–Fi 5 GHz ^1^	2400–2483 5150–5350 and 5470–5725

^1^ in systems operating in the TDD mode (*Time Division Duplex*): a single frequency band with a time-division of signal transmission to and from the base station is applied. ^2^ in systems operating in the FDD mode (*Frequency Division Duplex*): transmissions from the base station to the terminal (DL—*downlink*) and from the terminal to the base station (UL—*uplink*) are split into different frequency bands.

**Table 2:** Basic parameters of common broadband PEMs sensitive to RF EMF (according to manufacturers’ data) [19,20,21].

Type of PEM, Manufacturer (Country)	Parameter				
	Frequency Band, MHz	E-Field Measurement Range	Sampling Rate, s	Dimensions, cm	Weight, g
RadMan 2XT, Narda (Germany)	0.9–60,000	up to 200% of WELs	0.03 or 1	17 × 5 × 3.5	190
RadMan 2LT, Narda (Germany)	50–8000	up to 200% of WELs	1	17 × 5 × 3.5	190
WaveMon RF-8, Wavecontrol (Spain)	0.3–8000	up to 300% of WELs	1–3600	17 × 4.5 × 3.5	190
WaveMon RF-60, Wavecontrol (Spain)	0.1–60,000	up to 1000% of WELs	1–3600	17 × 4.5 × 3.5	190
EME Guard, MVG (France)	27–40,000	5–200 V/m	1–255	17 × 6 × 3.5	300
EME Guard Plus, MVG (France)	1–40,000	5–350 V/m	1–255	17 × 6 × 4	300

Note: applied workers exposure limits (WELs) were set by ICNIRP’1998 [8] and Directive 2013/35/EU [5].

**Table 3:** Basic parameters of common narrowband (selective, multi-channel) PEMs sensitive to RF EMF (according to manufacturers’ data) [21,22].

Type of PEM, Manufacturer (Country)	Parameter					
	Frequency Band, MHz	Number of Frequency Bands (Channels)	E-Field Measurement Range, V/m	Sampling Rate, s	Dimensions, cm	Weight, g
ESM-140, Satimo (France)	88–2480	8	0.001–0	0.5–10	12 × 4.5 × 3	87
EME SPY 121, Satimo (France)	88–2450	12	0.05–10	4–255	19 × 10 × 7	450
EME SPY 140, Satimo (France)	88–5850	14	0.01–5	4–255	17 × 8 × 6	410
EME SPY 200, MVG (France)	87–5875	20	0.05–6	2–255	17 × 8 × 6	440
EME SPY Evolution, MVG (France)	87–5875	max 20	0.05–6	2–255	18 × 7 × 5	520
ExpoM-RF3, Fields at Work (Switzerland)	87–5875	16	0.005–6	3–6000	16 × 8 × 5	320
ExpoM-RF4, Fields at Work (Switzerland)	50–6000	max 25	0.005–6 (optional 60)	3–6000	16 × 8 × 5	360

**Table 4:** Comparison of the dispersion of results for single and multi-point ball-probe measurement locations (ϕ50 mm E-field sensor) expressed by KX1bp ratio.

Measurement Points	KX1bp Range for Frequency						
	100 MHz	400 MHz	700 MHz	1000 MHz	1800 MHz	2600 MHz	3600 MHz
Head	0.56–3.21	0.59–1.05	0.94–1.47	0.93–1.07	0.72–1.15	0.96–1.23	0.89–1.07
Head + any 1 location	1.23–1.84	0.61–0.96	0.76–1.13	0.70–1.06	0.73–1.14	0.73–1.18	0.69–1.11
Head + any 2 locations	1.05–1.77	0.59–0.86	0.67–0.90	0.61–0.86	0.63–0.93	0.64–0.96	0.63–0.91
Head + any 3 locations	1.04–1.58	0.60–0.80	0.65–0.83	0.61–0.80	0.63–0.87	0.65–0.89	0.64–0.87
Head + any 4 locations	1.04–1.41	0.61–0.76	0.65–0.79	0.61–0.76	0.63–0.82	0.66–0.84	0.66–0.83
Head + any 5 locations	1.15–1.34	0.64–0.73	0.65–0.74	0.63–0.71	0.66–0.75	0.68–0.78	0.68–0.77
Head + 6 locations	1.24	0.68	0.69	0.66	0.70	0.72	0.72

**Table 5:** Comparison of the dispersion of results for single and multi-point small-sensor measurement locations (point E-field sensor) for 100 MHz frequency expressed by KX1ss ratio.

Measurement Points	KX1ss Range for 100 MHz Frequency, Point Probe at Various Distances to Body			
	5 mm	10 mm	15 mm	20 mm
Head	0.19–5.29	0.31–4.74	0.38–4.15	0.45–3.83
Head + any 1 location	1.58–2.47	1.42–2.24	1.36–2.09	1.32–2.00
Head + any 2 locations	1.25–2.33	1.16–2.17	1.13–2.04	1.10–1.96
Head + any 3 locations	1.16–2.01	1.10–1.89	1.09–1.79	1.08–1.73
Head + any 4 locations	1.14–1.79	1.09–1.70	1.08–1.63	1.07–1.58
Head + any 5 locations	1.29–1.59	1.25–1.52	1.23–1.47	1.20–1.43
Head + 6 locations	1.43	1.37	1.33	1.31

**Table 6:** Comparison of the dispersion of results for single and multi-point small-sensor measurement locations (point E-field sensor) for 3600 MHz frequency expressed by KX1ss ratio.

Measurement Points	KX1ss Range for 3600 MHz Frequency, Point Probe at Various Distances to Body			
	5 mm	10 mm	15 mm	20 mm
Head	0.56–1.33	0.95–1.18	0.91–1.34	0.87–1.49
Head + any 1 location	0.54–0.73	0.65–0.89	0.71–1.02	0.75–1.09
Head + any 2 locations	0.44–0.66	0.52–0.82	0.58–0.94	0.63–0.99
Head + any 3 locations	0.43–0.60	0.51–0.74	0.58–0.87	0.63–0.93
Head + any 4 locations	0.42–0.56	0.52–0.69	0.60–0.81	0.65–0.87
Head + any 5 locations	0.44–0.51	0.54–0.62	0.63–0.73	0.68–0.79
Head + 6 locations	0.46	0.56	0.66	0.72

## Data Availability

The original contributions presented in this study are included in the article. Further inquiries can be directed to the corresponding author.

## Abbreviations

The following abbreviations are used in this manuscript:

CW	Continuous Wave
DAB	Digital Audio Broadcasting
DL	Downlink
DVB-T	Digital Video Broadcasting-Terrestrial
FDD	Frequency Division Duplex
GTEM	Gigahertz Transverse Electromagnetic Mode
IoT	Internet of Things
LTE	Long Term Evolution
MMR	Minimum to Maximum Range
PEM	Personal Exposure Meter
RF EMF	Radiofrequency Electromagnetic Field
RFID	Radiofrequency Identification
RMS	Root Mean Square
SAR	Specific Absorption Rate
TETRA	Terrestrial Trunked Radio
TDD	Time Division Duplex
UL	Uplink
Wi–Fi	Wireless Fidelity
WiMAX	Worldwide Interoperability for Microwave Access
WLAN	Wireless Local Area Network
